# Supplementary material for: Performance of Salivary Extracellular RNA Biomarker Panels for Gastric Cancer Differs between Distinct Populations
Source: Cancers (Basel). 2022 Jul 26;14(15):3632. doi: 10.3390/cancers14153632 (PMC9331389; doi:10.3390/cancers14153632)
Supplement: Supplementary file 1 [file cancers-14-03632-s001.zip › cancers-1757035-supplementary.pdf]

Supplementary material

Supplementary Table S1. The list of mRNA biomarker candidates and primer sequences used for validation.

Gene Symbol	Accession No	Primer Sequence (5' to 3')	Amplicon Size* (bp)
PPL	NM_002705.4	OF:GGAAACAAAGGCAAATACAGC<br>OR:TGTGTCCACGATGTTCTTCTC<br>IF:CCGGAGCATCTCTAACAAGGA<br>IR:ACCTGGTCGGCATTCTTCTG	66
SEMA4B	NM_198925.2	OF:CAGCCTCTACCAGCCTCA<br>OR:CTGGAACTGGACTTGCTCA<br>IF:ATCCAGGACATCGAGGGAGC<br>IR:GTTGGTACAAAAGACGGGGAC	77
SPINK7	NM_032566.2	OF:CCTGCCCCATCACATACCTA<br>OR:AGAGCCTGGGATGATGAAGAT G<br>IF:CATCACCTATGGGAATGAATGTC<br>IR:TCCATCGTGAAGAAACTGAACT C	79
GAPDH #	NM_002046.4	OF:CAACAGCCTCAAGATCATCA<br>OR:CCATCACGCCACAGTTTC<br>IF:CCAACTGCTTAGCACCCCTG<br>IR:GGGCCATCCACAGTCTTCTG	112
ACTB #	NM_001101.3	CAGAGCCTCGCCTTTGCC<br>ATGCCGGAGCCGTTGTCG<br>CCTCGCCTTTGCCGATCC<br>GAGCGCGGCGATATCATCA	73

Abbreviations: O, outer; I, inner; F, forward; R, reverse.* The amplicon size is the nested PCR product size using IF+IR primers. # Saliva internal reference (SIR) gene.

Performance of miRNA and mRNA biomarkers for GC - Comparison between Korean and U.S. (MDA Anderson cohort)

Gene	GC (n = 100) vs non-GC control (n = 100) ΔCq			
	GC, mean (SD)	Non-GC, mean (SD)	P value[a]	AUC (95% CI)
ANXA1	−2.58 (2.07)	−3.36 (1.63)	0.008	0.61 (0.53, 0.69)
CD24	1.20 (1.90)	0.32 (1.66)	0.001	0.63 (0.56, 0.71)
CSTB	−2.83 (2.15)	−3.74 (1.79)	0.004	0.62 (0.54, 0.70)
EIF3G	6.98 (3.08)	7.08 (3.21)	0.945	0.50 (0.42, 0.58)
ERO1A	4.53 (2.07)	3.70 (1.96)	0.002	0.63 (0.55, 0.71)
KRT4	−2.28 (2.35)	−3.02 (2.00)	0.035	0.59 (0.51, 0.67)
KRT6A	−0.34 (2.34)	−1.21 (2.15)	0.001	0.63 (0.56, 0.71)
PPL	1.08 (2.23)	0.34 (2.20)	0.007	0.61 (0.53, 0.69)
RANBP9	4.26 (3.11)	3.56 (2.77)	0.157	0.56 (0.48, 0.64)
S100A10	2.21 (2.02)	1.55 (2.04)	0.006	0.61 (0.54, 0.69)
SEMA4B	11.47 (3.98)	10.57 (4.14)	0.149	0.56 (0.48, 0.64)
SPINK7	2.37 (2.72)	1.18 (1.98)	0.001	0.64 (0.56, 0.72)
MIR140-5p	1.54 (3.68)	−1.08 (3.27)	<0.001	0.70 (0.63, 0.78)
MIR374a	6.95 (5.69)	4.26 (4.59)	<0.001	0.65 (0.57, 0.73)
MIR454	4.61 (3.40)	3.14 (3.40)	0.003	0.63 (0.55, 0.70)
MIR15b	2.92 (3.52)	1.00 (3.42)	<0.001	0.65 (0.57, 0.72)
MIR28-5p	5.15 (4.17)	3.59 (3.94)	0.024	0.59 (0.51, 0.67)
MIR301a	8.46 (4.17)	6.95 (3.82)	0.01	0.61 (0.53, 0.69)

[a]All 15 biomarker candidates with $P < 0.05$ have q values (FDR-adjusted P values) of also <0.05.

Table 1. Performance of miRNAs & mRNAs in Korean cohort [Feng Li et al 2018].

mRNAs & miRNAs for GC	Control (n=49)	Case (n=51)	p-value	test
miR140	16.15 (2.67)	16.07 (2.62)	0.880 / 0.758	Wilcox / t-test
miR301	20.65 (2.45)	20.12 (2.60)	0.297 / 0.181	Wilcox / t-test
U6	15.37 (2.64)	15.53 (2.70)	0.776 / 0.809	Wilcox / t-test
miR197	16.20 (1.82)	16.19 (1.85)	0.998 / 0.796	Wilcox / t-test
dCTmiR140_U6	0.78 (2.41)	0.54 (1.74)	0.580 / 0.769	Wilcox / t-test
dCTmiR140_197	-0.05 (1.45)	-0.13 (1.24)	0.769 / 0.969	Wilcox / t-test
dCTmiR140_Both	0.41 (1.73)	0.24 (1.16)	0.578 / 0.779	Wilcox / t-test
dCTmiR301_U6	5.28 (2.34)	4.59 (1.74)	0.100 / 0.361	Wilcox / t-test
dCTmiR301_197	4.45 (1.49)	3.92 (1.27)	0.058 / 0.059	Wilcox / t-test
dCTmiR301_Both	4.91 (1.70)	4.29 (1.19)	0.038 / 0.136	Wilcox / t-test
geomeanBoth	15.74 (2.04)	15.83 (2.11)	0.841 / 0.856	Wilcox / t-test
PPL	21.72 (2.74)	22.01 (3.86)	0.997 / 0.673	Wilcox / t-test
SPINK7	23.98 (2.68)	24.58 (3.89)	0.533 / 0.369	Wilcox / t-test
SEMA4B	30.92 (2.81)	30.68 (3.13)	0.966 / 0.690	Wilcox / t-test
ACTB	17.96 (3.04)	18.39 (4.49)	0.905 / 0.578	Wilcox / t-test
GAPDH	25 (2.53)	24.83 (3.19)	0.845 / 0.765	Wilcox / t-test
PPL_ACTB	3.77 (2.09)	3.62 (2.08)	0.825 / 0.729	Wilcox / t-test
SPINK7_ACTB	-3.27 (2.5)	-2.81 (2.83)	0.403 / 0.393	Wilcox / t-test
SEMA4B_ACTB	12.94 (1.48)	12.32 (2.23)	0.315 / 0.112	Wilcox / t-test
PPL_GAPDH	-3.27 (2.5)	-2.81 (2.83)	0.403 / 0.393	Wilcox / t-test
SPINK7_GAPDH	17.96 (3.04)	18.39 (4.49)	0.905 / 0.578	Wilcox / t-test
SEMA4B_GAPDH	5.93 (1.98)	5.81 (2.19)	0.721 / 0.778	Wilcox / t-test

Table 2. Performance of miRNAs & mRNAs in U.S. MD Anderson cohort.

Supplementary Figure S1. Comparison of miRNA & mRNA biomarker performance for GC between Korean and U.S. MDA Anderson Cancer Center study groups.
